# Robust Event-Based Object Tracking Combining Correlation Filter and CNN Representation

**DOI:** 10.3389/fnbot.2019.00082

**Published:** 2019-10-10

**Authors:** Hongmin Li, Luping Shi

**Affiliations:** Department of Precision Instrument, Center for Brain-Inspired Computing Research, Tsinghua University, Beijing, China

**Keywords:** event-based vision, object tracking, dynamic vision sensor, convolutional neural network, correlation filter

## Abstract

Object tracking based on the event-based camera or dynamic vision sensor (DVS) remains a challenging task due to the noise events, rapid change of event-stream shape, chaos of complex background textures, and occlusion. To address the challenges, this paper presents a robust event-stream object tracking method based on correlation filter mechanism and convolutional neural network (CNN) representation. In the proposed method, rate coding is used to encode the event-stream object. Feature representations from hierarchical convolutional layers of a pre-trained CNN are used to represent the appearance of the rate encoded event-stream object. Results prove that the proposed method not only achieves good tracking performance in many complicated scenes with noise events, complex background textures, occlusion, and intersected trajectories, but also is robust to variable scale, variable pose, and non-rigid deformations. In addition, the correlation filter-based method has the advantage of high speed. The proposed approach will promote the potential applications of these event-based vision sensors in autonomous driving, robots and many other high-speed scenes.

## Introduction

Different from the traditional frame-based imager, event-based camera, or dynamic vision sensor (DVS) converts the temporal contrast of the light intensity into spatiotemporal, sparse event streams (Lichtsteiner et al., 2008; Serrano-Gotarredona and Linares-Barranco, 2013; Brandli et al., 2014). DVS has the advantages of low information redundancy, high dynamic range, and high speed in visual sensing, and has the potential applications in the high-speed scenes. Recently, DVS has been used for estimating the high-speed optical flow and intensity field (Kim et al., 2008; Bardow et al., 2016). A visual processing system based on event camera demands low energy consumption. The outputted events are represented in the form of Address-Event Representation (AER) (Boahen, 2000). AER is often utilized to model the signal of neural systems, like the retina using discrete time events to convey information, and other spike coded neural systems in living organisms.

**Figure 1:**
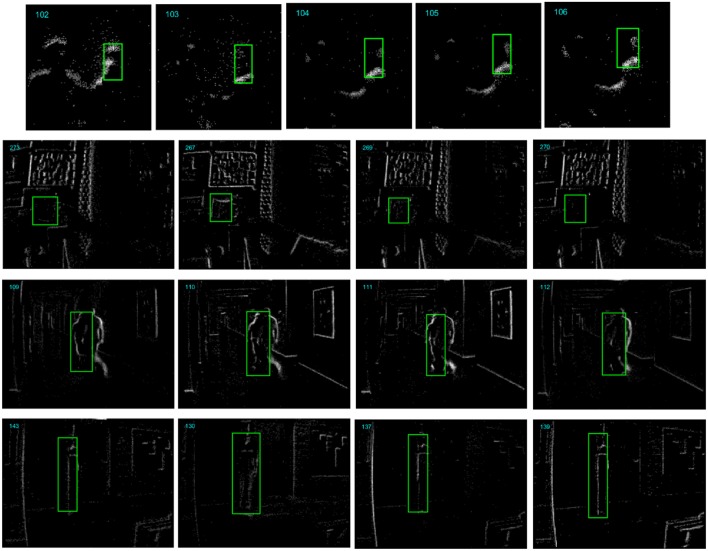
Some example reconstructed frames from several event-stream recordings (From top to bottom are the “Horse toy” from DVS128 sensor, “Vid_B_cup,” “Vid_J_person_floor,” and “Vid_E_person_part_occluded” from DAVIS sensor). The appearance of the target object (in bounding box with green line) changes obviously for the noise events, background texture, and randomness in event-generating of the pixels.

Visual tracking has a wide range of applications in the fields of autonomous driving, robot vision, trajectory analysis and so on. When an object is detected at a certain moment, it is often useful to track that object in subsequent recordings. Many works of object tracking based on the retina-inspired DVS sensors have been reported (Litzenberger et al., 2006; Conradt et al., 2009; Schraml and Belbachir, 2010; Drazen et al., 2011; Ni et al., 2012, 2015; Piatkowska et al., 2012; Zhenjiang et al., 2012; Saner et al., 2014; Zhu et al., 2017). However, event-based object tracking is challenging due to the significant appearance variations caused by the noise events, complex background textures, occlusion and randomness of event generating in each pixel circuit. Firstly, events stimulated by the contour and textures of the object are easy to be confused by events from the background objects. If the target event-stream object has a similar spatiotemporal shape with that of a background object, a tracker based on simple feature representation would be easily confused. Besides, the event-stream shape of the non-rigid object changes and deforms all the time, which demands a more discriminative feature representation. Figure 1 shows some successive reconstructed frames from several event-stream recordings. The ground-truth position of the target object is shown with a bounding box in each segment. From the pictures, we can see that the appearance of the target event-stream object changes obviously even between two adjoining segments, which demands a robust tracker for tracking the rapid changed appearance.

This paper presents a robust event-stream pattern tracking method based on correlation filter (CF) mechanism. Hierarchical convolutional layers of a convolutional neural network (CNN) are used to extract the feature representation from rate coding frame of event streams. The performance of the proposed method is evaluated on the DVS recordings of several complicated visual scenes. Among the recordings, three are captured by a DVS128 sensor (Lichtsteiner et al., 2008) by ourselves and the rest are from an event-stream tracking dataset (Hu et al., 2016) captured by a DAVIS sensor (Brandli et al., 2014). The results prove that the proposed method can successfully track the objects in some visual scenes with noise events, complicated background textures, occlusion, and intersected trajectories. This is because we use features from multiple CNN layers to represent the appearance of the target object which combines semantics that are robust to significant appearance variations and spatial details that are effective for precise localization.

### Related Works

Object tracking methods based on event cameras can be classified into two categories. The first category is the event-driven mechanism in which each incoming event is processed and determined whether it belongs to the target object. In Litzenberger et al. (2006) implemented a continuous clustering of AER events and tracking of clusters. Each new event was assigned to a cluster based on a distance criterion and then the clusters weight and center position was updated. In addition, point cloud method is also introduced to model the event-stream object. In Ni et al. (2015) proposed an iterative closest point based tracking method by providing a continuous and iterative estimation of the geometric transformation between the model and the events of the tracked object. In Ni et al. (2012), the authors applied the iterative closest point tracking algorithm to track a microgripper position in an event-based microrobotic system. One disadvantage of these kinds of methods is that noise events occur will cause the tracker to make a wrong inference. Adding noise event filtering modules to the tracking system will unavoidably filter many informative events while increase the computational complexity of the system. In addition, although these event-based sensors are based on the event-driven nature, it is still a difficult task to recognize an object from each single event. The second category is based on feature representation of the target object. In Zhu et al. (2017), the authors proposed a soft data association modeled with probabilities relying on grouping events into a model and computing optical flow after assigning events to the model. In Lagorce et al. (2014), proposed an event-based multi-kernel algorithm, and various kernels, such as Gaussian, Gabor, and arbitrary user-defined kernels were used to handle the variations in position, scale and orientation. In Li et al. (2015), the authors prosed a compressive sensing based method for the robust tracking based on the event camera. The representation or appearance model of event-stream object is based on features extracted from the multi-scale space with a data-independent basis and employs non-adaptive random projections that preserve the structure of the feature space of objects.

The core of most modern trackers is a discriminative classifier to distinguish the target from the surrounding environment. In computer vision, CF based methods has enjoyed great popularity due to the high computational efficiency with the use of fast Fourier transforms. In Bolme et al. (2010) learned a correlation filter over luminance channel the first time for real-time visual tracking, named MOSSE tracker. In Henriques et al. (2012, 2015), a kernelized correlation filter (KCF) is introduced to allow non-linear classification boundaries. Nowadays, features from convolutional neural network (CNN) are used to encode the object appearance and achieved good performance (Danelljan et al., 2015; Ma et al., 2015). In Danelljan et al. (2015), the authors proposed a method combining activations from the convolutional layer of a CNN in discriminative correlation filter based tracking frameworks, achieving a superior performance by using convolutional features compared to standard hand-crafted feature representations. They also show that activations from the first layer provides superior tracking performance compared to the deeper layers of the network. In Ma et al. (2015), they exploit the hierarchies of convolutional layers as a non-linear counterpart of an image pyramid representation and these multiple levels of abstraction to improve tracking accuracy and robustness. They demonstrate that representation by multiple layers of CNN is of great importance as semantics are robust to significant appearance variations and spatial details are effective for precise localization. Although feature-based methods show robustness and real-time capability, the most serious defect is that such algorithms should accumulate the events in a time window and then perform feature extraction. Then the length of the time window may be different under different scenes.

## Methodology

### Temporal Contrast Pixel

The pixel of the DVS sensor is a type of temporal contrast pixel which only responds to the temporal contrast of the light intensity in the scene and generates a temporal event whenever the brightness change exceeds a pre-defined threshold. Each event is a quadruple (*x, y, t, p*), where (*x, y*) denotes the positions of the pixel, *t* denotes the time when the event is generated, the polarity *p* = 1 denotes the increasing brightness and *p* = −1 denotes the decreasing brightness. This temporal contrast pixel has the advantage of high dynamic range because it needs not to respond to the absolute light intensity. The time stamp of each event has the temporal resolution of microsecond. Then the DVS sensor is suitable to capture the dynamic scenes with high-speed changes.

In this work, we use event-stream recordings from both DVS128 and DAVIS sensors to evaluate the performance of the proposed method. Both sensors are based on the same event-generating mechanism. As the name of the sensor shows, DVS128 has the spatial resolution of 128 × 128. DAVIS is a new retina-inspired, event-based vision sensor with the spatial resolution of 240 × 180.

Although these kinds of sensors are based on an event-driven nature, it remains a difficult task to recognize an object from a single event. Many works have accumulated the event stream into multiple segments on which to extract feature for information processing, such as the event-stream object display in jAER open-source tool. There exist two accumulating methods, i.e., hard events segmentation (HES) and soft events segmentation (SES). HES divides the event flow into segments using fixed time slices or fixed number of events. Different from HES, SES adaptively obtains the segments according to the statistical characteristics of the events based on an event responding neuron, such as the leaky-integration-firing neuron.

For comparison, we segment the event streams into the same number of segments with the items of the groundtruth. Rate coding is utilized to encode the visual information of the event-stream object. Intuitively, each pixel value is represented as the number of events generated by this pixel within the segment. Event rate of the temporal contrast pixel can be represented as follows,

(1) $$\begin{array}{r} Rate\left(t\right)\approx \frac{TCON\left(t\right)}{\theta }=\frac{1}{\theta }\frac{d\ln\left(I\right)}{dt} \end{array}$$

where TCON represents the temporal contrast, and *I(t)* is the photocurrent. Within a time window, the physical meaning of the number of events of a pixel represents the frequency of which the temporal intensity change exceeds the threshold. In rate coding, the serious temporal noise of the events is suppressed by integrating the events of each pixel in the segment.

### Correlation Filter Framework

Generally, a CF tracker learns a discriminative classifier and finds the maximum value of the correlation response map as the estimation of the position of the target object. The resulting classifier is a 2-dimensional correlation filter which is applied to the feature representation. Circular correlation is utilized in CF framework for efficiently train. Multi-channel feature maps from multiple layers of a deep CNN are used as representation of rate-coding event-stream object. Feature maps of the *l*-th layer is denoted as *x*^*l*^ with the size of H × W × C, where H, W and C denote the width, height, and channel number, respectively. The correlation filter *f*_*t*_ has the same size with the feature maps in the current event frame *t*. In CF framework, the correlation filter is trained by solving a linear least-squares problem as follows,

(2) $$\begin{array}{r} \underset{f_{t}}{w=\arg\min}\underset{h,w}{\sum }{∥f_{t}\cdot {x^{l}}_{h,w}-y_{h,w}∥}^{2}+\lambda {\left\|f_{t}\right\|}_{2}^{2} \end{array}$$

where $x_{h,w}^{l}$ demotes the shifted sample. hε{0,1,2,…,H-1}, wε{0, 1, 2, …, *W*-1}. *y*_*h, w*_ is the Gaussian function label, and where λ is a regularization parameter (λ > 0). $y_{h,w}=\exp\left(-\frac{{\left(h-H/2\right)}^{2}+{\left(w-W/2\right)}^{2}}{2\sigma^{2}}\right)$, where σ is the kernel width.

The minimization problem in (2) can be solved in each individual feature channel using fast Fourier transformation (FFT). We use the capital letters as the corresponding Fourier transform of the signal. The learned correlation filter in frequency domain on the *c-th*, cε{1, 2, …, C} channel is as follows,

(3) $$\begin{array}{r} F^{c}=\frac{Y⊙{\bar{X}}^{c}}{\sum_{i=1}^{C}X^{i}⊙{\bar{X}}^{i}+\lambda } \end{array}$$

where the operation ⊙ denotes the element-wise product, *Y* is the Fourier transformation form of y = {y_h, w_ | hε{0, 1, 2, …, *H*-1}, wε{0, 1, 2, …, *W*-1}}, and the bar means complex conjugation. z^l^ with size of H × W × C represents the feature maps of the l-th layer of the neural network. The l-th correlation response map of size H × W can be calculated as follows,

(4) $$\begin{array}{r} r^{l}=\xi^{-1}\left(\overset{C}{\underset{c=1}{\sum }}F^{c}⊙{\bar{Z}}^{c}\right) \end{array}$$

where the operation ξ ^−1^ denotes the inverse FFT transform. The position of maximum value of the correlation response map r^*l*^ is used as the estimation of the target location on the l-th convolutional layer.

### Representation Based on Convolutional Neural Network

**Figure 2:**
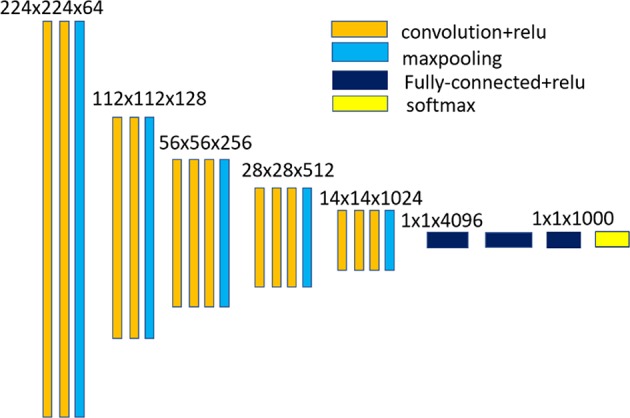
Network architecture of the VGG-Net-16.

In this paper, hierarchical convolutional feature representation is used for encoding the appearance of the event-stream object. An imagenet-pretrained 16-layer classification network (VGG-Net-16)^1^ implemented based on the MatConvNet library is used in our method for feature extraction. Figure 2 shows the network architecture of the VGG-Net-16. Hierarchical feature representation are obtained with the CNN forward propagation. Event streams in a short duration are integrated into a rate coding map which is taken an the input of CNN. As the input of the original VGGNet are three-channel, we set each channel of the input layer equal to the rate coding map. As the pooling operation would reduce the spatial resolution with increasing depth of convolutional layers, we first remove the layers higher than the conv3_3 layer and the output of conv1_1, conv2_2, and conv3_3 are taken as the feature. Multiple convolutional layers are combined to encode the changed appearance of the event-stream object. **Table 2** shows the spatial size and channels of the feature maps of the input layer, and three different convolutional layers. Instead of resizing the size of the input rate coding maps to equal the size of the input layer of CNN (i.e., 224 × 224) in Ma et al. (2015), we use the resulted model parameters of each layer to perform convolutional operation on the original input. In this work, we test some hierarchical composition of different convolutional layers for feature representation and found that the representation of combination of multiple layers of conv1_1, conv2_2, and conv3_3 could achieved a satisfactory tracking performance.

## Experimental Results and Discussion

In this section, a series of experiments on several event-stream recordings are presented. The metric used in this paper is the center location error. We labeled the ground-truth position of tracked object manually. Section 4.1 introduces the event-stream tracking dataset on which we perform the tracking experiments. In section 4.2, we evaluate the influence of two hyper-parameters. Section 4.3 presents the tracking speed over different convolutional layers of CNN. In section 4.4, we compare the performance of the proposed method with some other event-stream tracking methods.

### Event-Stream Recordings

Eight event-stream recordings with labeled ground truth data are used to evaluate the proposed method. Among them, three recordings were captured by a DVS128 sensor by ourselves, and the rest are from an event-stream tracking dataset captured by a DAVIS sensor.

*A. Three DVS128 recordings*^2^ Three event-stream recordings of three different scenes were captured with a DVS128 device. These recordings have the same spatial resolution of 128 × 128. We divided each recording into multiple segments and in each segment, we label the position and the size of the target object with bounding boxes. The first recording (“Digit3”) is a scene containing several digits. The task is to track the digit3 in the event streams. This recording has a time range of about 38.8 s which is divided into 767 segments. The second recording (“Horse toy”) is a moving human with a horse toy in his hand. The task is to track the horse in the event streams. This recording has a time duration of about 17.3 s and is divided into 347 segments. The appearance of the toy changed quickly with much rotation and deformation in the recording. The third recording (“Human face”) has a duration of about 17.1 s and is divided in 343 segments. This task is to track the face of a human. The human face moved quickly with rotation and deformation and the appearance of the event stream changed all the time, which make the task difficult.

*B. DAVIS recordings*^3^ Yuhuang Hu et al. (2016) proposed an event-stream tracking dataset with a DAViS240C camera which has a spatial resolution of 240 × 180. In some tracking sequences of this tracking dataset, the target objects are still, or cannot be distinguished from the background. We chose five recordings from this dataset and re-labeled the bounding boxes of the target objects because the provided bounding boxes in the dataset seem to have a little shift compared to the accurate positions of the target objects. These chosen five recordings are “Sylvestr,” “Vid_B_cup,” “Vid_C_juice,” “Vid_E_person_part_occluded,” and “Vid_J_person_floor,” and are divided into 1344, 629, 404, 305, and 388 segments, respectively.

*C. Challenges in each recording*. We list the challenges in each recording as show in Table 1. Seven challenges are taken into consider, including the noise events, complicated background, occlusion, intersected trajectories, deformation, scale variation and pose variation. One or several challenges are contained in each recording.

**Table 1:** Challenges of each recording.

**Challenge**	**Noise event**	**Complicated background**	**Occlusion**	**Intersected trajectories**	**Deformation**	**Scale variation**	**Pose variation**
Digit3	1	0	0	0	0	1	0
Horse toy	1	0	0	0	1	1	1
Human face	1	0	0	0	1	1	1
Sylvestr	1	0	0	0	1	1	1
Vid_B_cup	1	1	0	0	0	0	1
Vid_C_juice	1	1	0	1	0	1	0
Vid_person_part_occluded	1	1	1	0	0	0	0
Vid_J_person_floor	1	1	1	1	1	1	1

*If the recording (in the row) has this challenge (in the column), then the corresponding value is set to 1, otherwise 0*.

### Robustness to Hyperparameters

The proposed event-stream pattern tracking method requires the specification of two hyparameters, i.e., the event number in each segment and the convolutional layers for feature representation. To investigate the influence of these two hyperparameters, we performed a series of experiments on several event-stream recordings.

**Figure 3:**
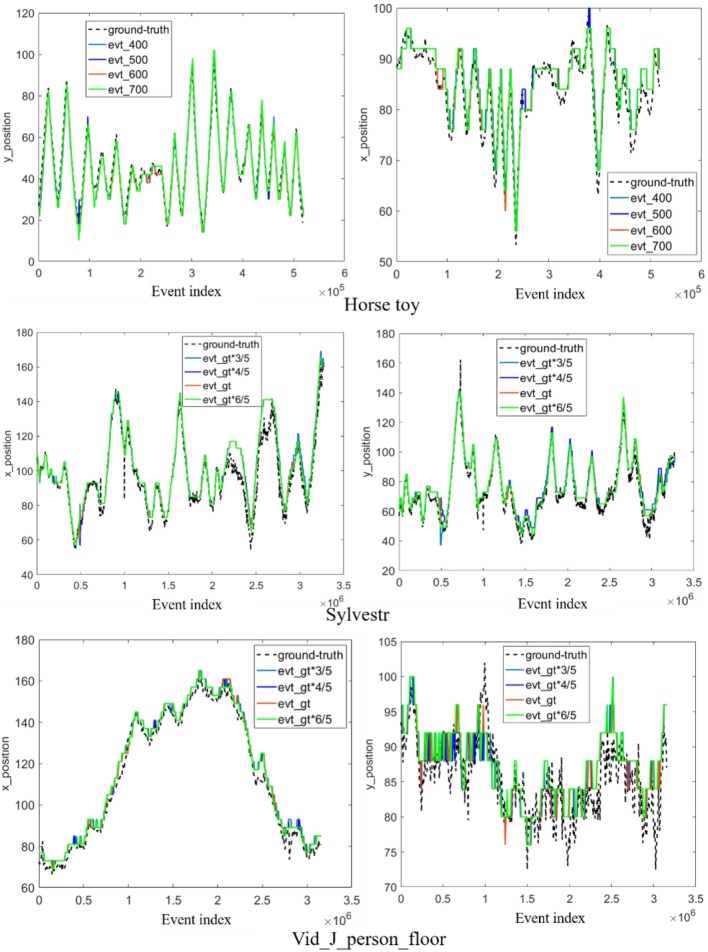
Tracking performance under different event number per time bin on three event-stream recordings (from top to bottom are “Horse toy,” “Sylvestr,” and “Vid_J_person_floor” scenes, respectively). The x (left) and y (right) positions of the center of the tracker over the time are displayed. We use the index of event to represent the time coordinate, and the * mean multiplication.

*A. Event number in each segment*. We investigate the influence of the number of events in each segment on several recordings, including the “Horse toy” from DVS128 sensor and the “Sylvestr,” “Vid_J_person_floor” from DAVIS sensor. Figure 3 shows the tracking trajectory of the proposed tracker under different event number in each segment. The change of the x position and y position of the center of the tracker along time are shown. As the number of segments would be different from that of the groundtruth with a different event number in each segment, it would be impossible to compare the location of the trackers when both have different number of segments. Then we use the index of the events to represent the time coordinate. We plot the curve of the *x* position and *y* position of the tracker over the index of events. Results show that the proposed method is robust to the number of events in each segment by comparing the degree of proximity of the trajectory of the tracker and the groundtruth. The effectiveness of the proposed method is owing to the discriminative feature representation transferred from the multiple layers of a pre-trained CNN on the computer vision task. The proposed method has many potential applications in many the high-speed scenes with less events in each time window.

**Figure 4:**
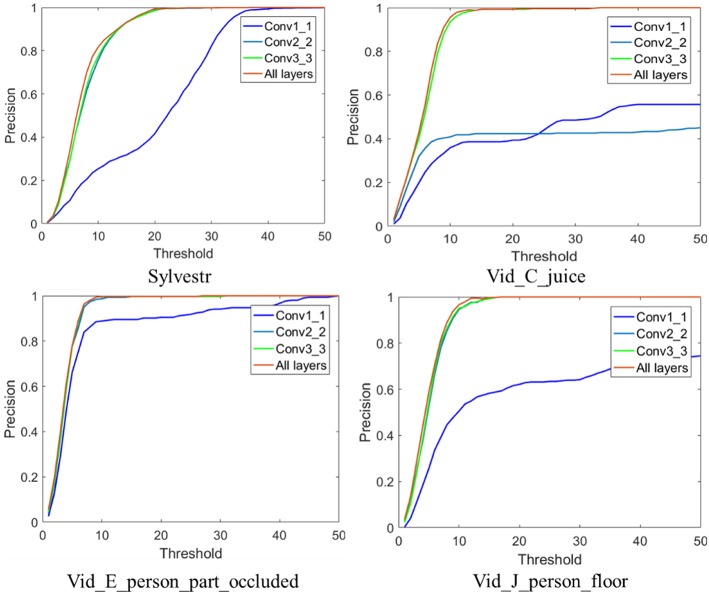
Tracking performance with different feature representation from different convolutional layers of the employed CNN.

*B. Feature map from different layers in the CNN*. We investigate the influence of different convolutional layers of VGG-Net-16 model on the tracking performance. Feature representations with four kinds of combination of convolutional layers, i.e., the Conv1_1 convolutional layer, the Conv2_2 layer, the Conv3_3 layer, and the composition of the three convolutional layers were evaluated. Table 2 shows the spatial size and dimensionality of the feature maps of the input layer, and three different convolutional layers. All the DAVIS recordings (“Sylvestr,” “Vid_B_cup,” “Vid_C_juice,” “Vid_E_person_part_occluded,” and “Vid_J_person_floor”) are used in this test. For the “Vid_B_cup” scene, the tracking would fail when a single convolutional layer or a composition of two layers (Conv1_1 or Conv2_2 or Conv3_3) is used. In “Vid_B_cup,” the target cup is moved over a complicated background, then the events from the target object are very easy to be mixed up with the events from background. Figure 4 shows the metric results on the rest four event-stream recordings with different convolutional layers. Feature representation from the higher convolutional layers results in better tracking performance. For the more complicated scenes with complex background, such as the “Vid_B_cup,” combination of hierarchical feature representation from multiple convolutional layers is required for effective object tracking. This is because the feature representation from multiple convolutional layers combines the low-level texture features and high-level semantic features and can handle the rapid change of the appearance of the target object. While for the relatively simple scenes with less noise events and simple background textures, less and lower convolutional layers result in effective tracking with a higher speed.

**Table 2:** Spatial size and channels of the feature maps from three different convolutional layers of the employed network.

	**Input**	**Conv1_1**	**Conv2_2**	**Conv3_3**
Spatial size	M × N	M × N	M/2 × N/2	M/4 × N/4
Channels	3	64	128	256

*Input layer denotes the input rate coding map, after the necessary preprocessing steps*.

### Tracking Speed Under Different Layers

In this section, we investigate the tracking speed of the proposed method on several recordings. The speed is measured over different convolutional layers of the employed CNN. Table 3 shows the results of tracking speed on five DAVIS recordings. We measure the tracking speed using the unit of segments per second which is similar to the frame per second in the computer vision tracking. In the experiments, a PC machine with Intel(R) Core(TM)i5-7300HQ CPU @ 2.5 GHz is used. We did not present the measurement result on some convolutional layers in some event-stream recordings (such as the Conv1_1, and Conv2_2 in the “Vid_C_ juice” scene) which have failed in the tracking of the corresponding target object.

**Table 3:** Tracking speed under different convolutional layers of the employed network.

**Event recordings**	**Conv1_1**	**Conv2_2**	**Conv3_3**	**All**
Sylvestr	114.1	68.8	38.2	30.1
Vid_C_juice	–	–	37.8	29.8
Vid_E_person_part_occluded	–	68.5	37.9	29.2
Vid_J_person_floor	–	68.3	38.1	29.0
Vid_B_cup	–	–	–	29.3

*The tracking speed is measured by the segments per second. We measure the tracking speed on the DAVIS recordings*.

Intuitively, high-level representation requires more computational operations, which leads to the decrease of the tracking speed. In the proposed method, the precision and the speed are a tradeoff. In the simple scenes, such as monitoring an object with a fixed sensor, low-level convolutional layers are enough for effectively tracking with high speed. In other complicated scenes such as complicated background textures or moving DVS sensor, high-level convolutional layers are demanded for accurate tracking, which limits the tracking speed. In fact, due to the high computational efficiency in the frequency domain of the CF mechanism, the proposed method achieved relatively high tracking speed even using high-level feature map.

### Comparison With Other Methods

In this section, we compare the performance of the proposed method to several other event-stream pattern tracking methods. We perform the experiments on all the eight event-stream recordings. In the first place, we introduced the four other tracking methods as follows:

#### A. Three Tracking Methods in jAER Software

These three methods are based on the three event filters in jAER source available within the jAER sourceforge repository, including “Rectangular Cluster Tracker,” “Einstein Tracker,” and “Median Tracker,” respectively. Since the three methods have failed in the more complicated scenes, we only provide the tracking results of the three methods on the three simple scenes captured by DVS128 sensor.

#### B. Compressive Tracking-Based Rate Coding Feature

This is a feature-based event-stream tracking method based on compressive sensing and has achieved good performance on some simple scenes captured by DVS128. The compressive tracking learns a classifier on the compressive coding of multi-scale haar-like feature extracted on the rate coding map. We provide the results of this method on all the event-stream recordings for comparison with the proposed method.

**Figure 5:**
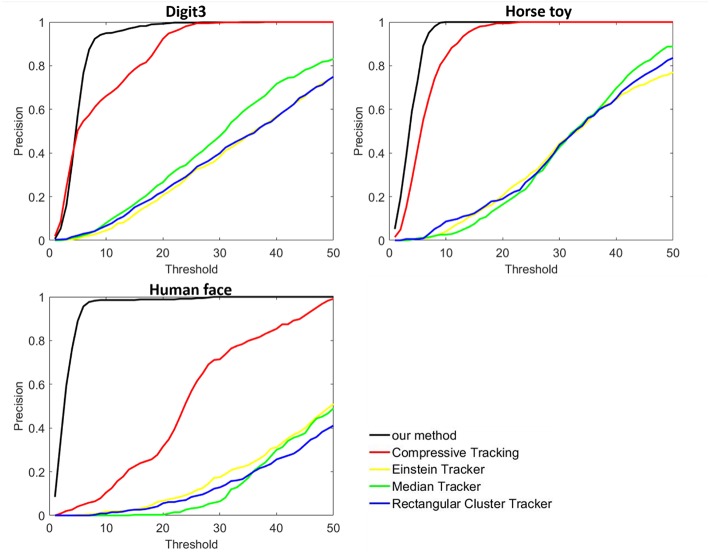
Tracking results on three event-stream recordings captured by DVA128 sensor (from left to right are Digit3, Horse toy, and Human face, respectively). Comparison were done with four other tracking methods.

Figure 5 shows the comparison results of the proposed method with all the four methods on the DVS128 recordings. Center location error is used to measure the tracking performance. Results show that the proposed tracking method achieves the best performance. Three tracking methods in jAER software show poor tracking performance. The event-driven jAER methods often failed to assign each event to the accurate position or cluster of the target object. Compared to the compressive tracking method, the proposed method achieved better performance owning to the more discriminative CNN feature presentation than the haar-like feature in the compressive tracking.

**Figure 6:**
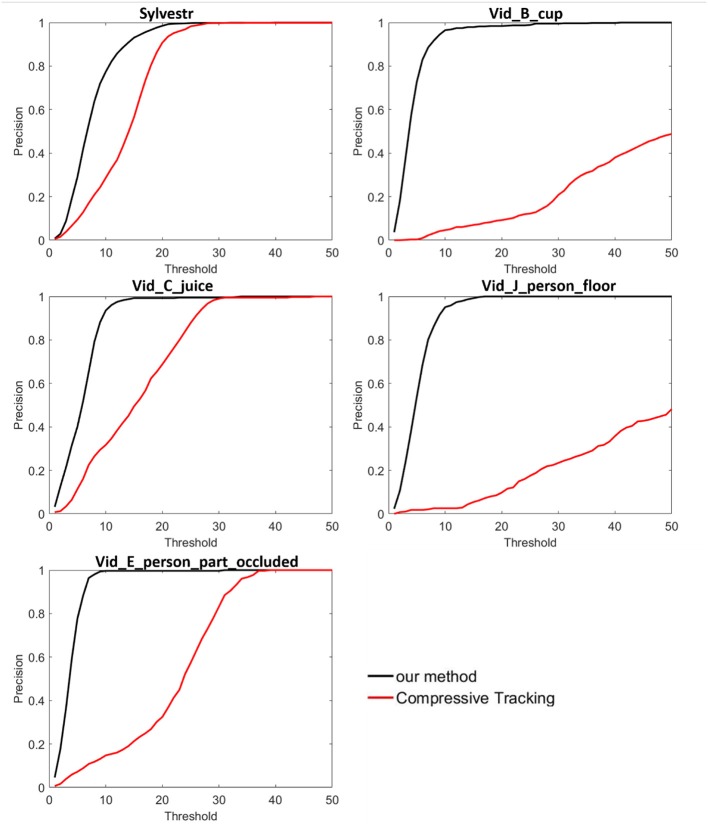
Tracking results on five event-stream recordings captured by DAVIS sensor, compared with the compressive sensing-based tracking methods.

On the five DAVIS recordings, we only compared the tracking performance of the proposed method with the compressive tracking method because the three tracking methods in jAER software fail to track the target object in the more complicated scenes. Figure 6 shows the tracking location precision of the two methods. The proposed method achieved better performance on the five DAVIS recordings. Especially in the “Vid_B_cup” scene where many objects in the background have the same shape with the target object “cup,” the proposed tracker can track the target object correctly. Simple hand-crafted feature representation cannot handle many complicated scenes with noise events, complicated background textures, rapid changed appearance, and occlusion. By combining the low-level texture features and high-level semantic features, the feature representation from multiple convolutional layers can handle the complicated scenes very well. Results demonstrate that the proposed method is robust to many challenging visual scenes with better tracking performance than other methods.

**Figure 7:**
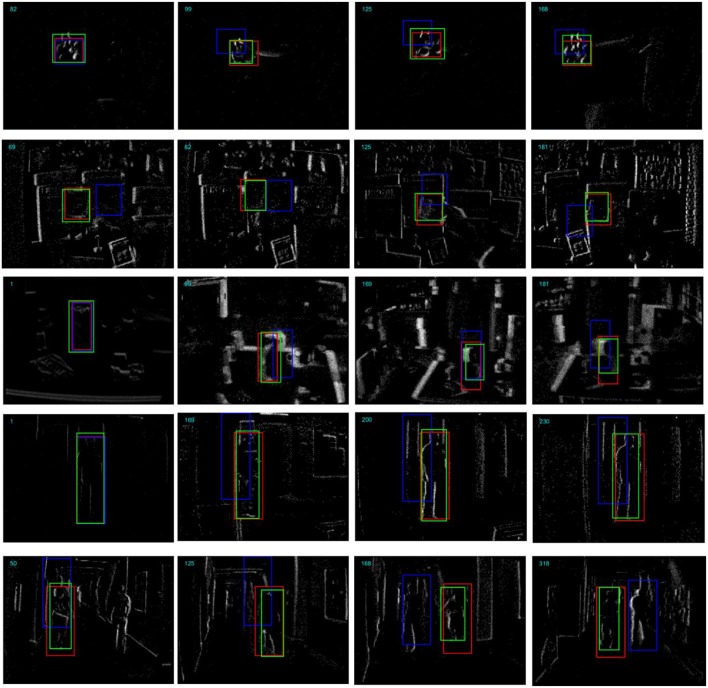
Comparison of the proposed tracking method with the compressive sensing based method on example segments from the five DAVIS recordings. From top to bottom are the “Sylvestr,” “Vid_B_cup,” “Vid_C_juice,” “Vid_E_person_part_occluded,” and “Vid_J_person_floor,” respectively. The bounding boxes with green, red, and blue color represent the groundtruth, our proposed tracker and the compressive sensing based tracker, respectively.

Figure 7 shows some tracking examples by integrating the events into reconstructed frames. In the tracking process, we did not change the scale of the tracker. The proposed tracker tracked the target object with high location precision while the compressive sensing-based tracker drifts very easily. Even with complicated background and occlusion, our tracker achieved very high tracking precision.

## Conclusion

In this work, we proposed a robust event-stream object tracking method based on the CF tracking mechanism. Our method overcomes some challenges in the event-stream tracking, such as the noise events, chaos of the complex background texture, occlusion, and randomness of event generating in the pixel circuit. Rate coding is used to encode the visual information of the target event-stream object. Correlation response map is computed on the feature representation from the hierarchical convolutional layers of a pre-trained deep CNN.

The proposed method shows good performance in many complicated visual scenes. Compared with other feature-based methods, our method is more robust in many visual scenes with noise and complicated background textures. Compared with other event-driven method, the proposed method has real-time advantage on event streams with large number of events. To utilize each single event for tracking and updating the appearance of the target object without segment reconstruction is a more interesting and challenging task which has lied in the heart of current event-based vision research and will be explored in the future. In event-driven tracking tasks, how to suppress the noise events is an import step for correct tracking as the noise events will lead the tracker to make wrong estimation.

## Data Availability Statement

Publicly available datasets were analyzed in this study. This data can be found here: https://figshare.com/s/70565903453eef7c3965; https://figshare.com/s/70565903453eef7c3965.

## Author Contributions

HL proposes the algorithm and design the experiment setup. Besides, data analysis and classification metric are also done by HL. LS supports the research of the neuromorphic vision and contributes the principle of event-based object tracking.

### Conflict of Interest

The authors declare that the research was conducted in the absence of any commercial or financial relationships that could be construed as a potential conflict of interest.

## Footnotes

^1^The model can be download from: https://pan.baidu.com/s/1QWl6zw4DpMSCDg2X-Mb7mAA

^2^The DVS128 recordings and the groundtruth, named “DVS recordings and groundtruth.zip” can be download from: https://figshare.com/s/70565903453eef7c3965

^3^The DAVIS recordings and the groundtruth, named “DAVIS recordings and groundtruth.zip” can be download from: https://figshare.com/s/70565903453eef7c3965
